# Anterolateral approach for benign proximal femoral lesions among Indian children

**DOI:** 10.6026/973206300222980

**Published:** 2026-05-31

**Authors:** Aditya Jain, Sitanshu Barik, Anil Agarwal, Lokesh Sharma, Yogesh Patel, Varun Garg

**Affiliations:** 1 Department of Orthopedics, Chacha Nehru Bal Chikitsalaya, New Delhi, India; 2 Department of Orthopedics, All India Institute of Medical Sciences, Nagpur, India

**Keywords:** Neck femur, lytic lesion, benign lesion, fibula, anterolateral approach hip

## Abstract

Benign lytic lesions of the proximal femur in children cause pain, deformity, and pathological fractures, posing major challenges for bone stability and healing. Recent studies report pathological fractures in up to 32% of pediatric proximal femoral cysts and recurrence rates of about 17% after curettage and filling. Similarly, multicenter data from the European Paediatric Orthopaedic Society (EPOS) show failure rates of 36-43% in aneurysmal bone cysts of the proximal femur, highlighting the difficulty of achieving durable healing. Therefore, it is of interest to present the results of treating lytic lesions of the proximal femur in children through an anterolateral approach using curettage and non-vascularized fibular grafting. Hence, this retrospective study included 14 skeletally immature patients (mean age 7.1 years). The hip joint was exposed using the standard anterolateral approach, and fibula was harvested through a posterolateral incision. Aneurysmal bone cysts were the most common diagnosis (57.1%). Five patients (36%) required implant stabilization. At final follow up, all patients showed radiological healing at a mean of 4 months, with a mean Harris Hip Score of 78.8. Thus, the anterolateral approach with fibular grafting provides reliable exposure, facilitates bone healing, and allows implant use while preserving the lateral cortex in pediatric proximal femoral lesions.

## Background:

Benign lesions of bone are a group of tumor or tumor-like conditions that are primarily lytic, with the proximal femur being one of the most common sites of occurrence [1]. Lesions of the proximal femur in children can present with pain, deformities, gait disturbances, and, most importantly, pathological fractures [2]. Managing these lesions poses a significant challenge regarding bone loss and stabilization options. Challenges also remain to make the child upright and ambulatory by promoting active bone healing. The osseous component of the hip region includes flat bones of the pelvis along with tubular bone of the proximal femur which comprises the femoral head, neck and greater trochanter [3]. These osseous elements are particularly rich in hematopoietic tissue [4]. With the ensuing high metabolism and blood flow in these areas, they are one of the common sites of tumors, both benign and metastatic. Intralesional curettage and grafting are the frequent treatments for these lesions as they are both diagnostic and curative [5]. The approach to the hip requires careful planning as curettage further weakens the cortices surrounding the lytic zone. Although more straightforward, the lateral approach makes implant use difficult as most implants for fixation of the proximal femur mandate an intact lateral cortex [6]. We have been practicing the extended version of Watson Jones's approach to the hip for lesions of the proximal femur to overcome this problem. Therefore, it is of interest to present the treatment results of lytic lesions of the proximal femur through an anterolateral approach using curettage and non-vascularized fibular grafting in children.

## Methods:

## Study details:

Based on records from August 2013 to July 2021, this retrospective study was conducted in a tertiary teaching pediatric hospital. It was part of more extensive research investigating outcomes after non-vascularized fibular bone grafting. Ethical clearance was obtained for the research. The study included skeletally immature patients with lytic benign proximal femur lesions. Patients with incomplete data records and follow-ups of less than 24 months were excluded.

## Diagnosis:

A multi-pronged strategy was used to arrive at the probable diagnosis. Complete history, physical examination, laboratory tests and radiographs of the pelvis and femur were done routinely. In selected patients, advanced imaging like a CT or MRI scan was done to assess the status of the bone, the surrounding soft tissues, and the lesion.

## Hip procedure:

The hip joint was exposed by a surgical team using the standard anterolateral approach between the abductors and vastus lateralis posteriorly and tensor fascia lata anteriorly in the supine position [7]. Once the capsule was visualized, a T-shaped capsulotomy was done to expose the femoral neck and part of the femoral head. The vastus lateralis was slit in the middle, and the posterior part of the muscle, along with the hip capsule, was reflected inferiorly as a flap. Homan retractors were used to assist in visualizing the neck region and proximal femur distal to the trochanter. A bone chisel was used to create a fenestration window on the anterior aspect of the femoral neck. A curette removes pathological tissue, including bone and soft tissues. These tissues were sent for histopathological examination. High-speed burr was used in patients where dense sclerotic bone was encountered. If one or more cortices of the femur were found fractured post-curettage, the stabilization was achieved using an appropriate implant. The cavity was subsequently packed with match stick fibular bone grafts. Additional tricalcium phosphate synthetic grafts were used where the cavity was incompletely filled even after using both fibulae. Postoperatively, the region was protected in a hip spica for 2 months. Following removal of spica and radiographic confirmation of healing, the child initiated partial weight bearing followed by full weight bearing within 4-6 weeks.

## Fibula harvest:

The fibula was harvested using a posterolateral incision over the leg [8]. The fibula was exposed by retracting the soleus muscle posteriorly and the peroneal muscle anteriorly. A single clean incision was made on the periosteum down till the cortex along the length of the fibula needed to be excised. Careful elevation of the periosteum was done circumferentially to leave the periosteal sleeve intact on all sides. Once the periosteum had been elevated along the desired length, osteotomy was done at the proximal and distal ends to harvest the diaphyseal fibula. The bone was removed, and the periosteal sleeve was sutured loosely, followed by layered closure. The harvested fibula/ fibulae were converted into match sticks approximately 3 cm long and the curetted cavity packed.

## Outcome measures:

The medical records provided demography, clinical symptoms, signs, and diagnosis details. The outcome was assessed by the timing of radiological healing at the proximal femur and the fibular harvest site. Radiological healing was defined as occlusion of lytic areas and thickening of the four bone cortices. At the final follow-up, the function of the hip was assessed using the Harris Hip Score. Any complication or uneventful occurrence was noted.

## Results:

Fourteen children, all males, with a mean age of 7.1 years (range, 3.1 to 12.5 years) were included in the study. The left proximal femur (10/14, 71.4%) was involved in most patients. None of the patients had any bilateral involvement. One of the patients had a polyostotic involvement resulting from fibrous dysplasia apart from the proximal femur lesions. The mean follow-up available was 3.2 years (2 to 6 years). Aneurysmal bone cyst(8/14, 57.1%) was the most typical diagnosis (Table 1). One of the patients had an aneurysmal cyst secondary to an eosinophilic granuloma. The pathological fracture was noted pre-operatively in two patients with a diagnosis of polyostotic fibrous dysplasia and aneurysmal bone cyst. Implants were used to stabilize the bone in 36% (5/14) of the patients. The various implants used were cannulated cancellous screws, rush rods, and dynamic hip screws. At the final follow-up, the patients had a mean Harris Hip Score of 78.8 (range 68 to 92). All hips were painless, and none had difficulty squatting or sitting cross-legged. One patient required revision surgery due to the recurrence of non-ossifying fibroma. All the patients had radiological healing at the proximal femur at a mean of 4 months (range, 2 to 8 months) after the index surgery. Radiological healing was noted after 4 months of the revision grafting procedure in this case of recurrence. One patient did not regenerate the fibula at the last follow-up of 2.6 years and is currently under observation. Recurrence was noted in a non-ossifying fibroma patient who underwent revision intralesional curettage and grafting and healed uneventfully thereafter. Implant loosening was noted in the patient with eosinophilic granuloma with a secondary aneurysmal bone cyst and was treated with implant removal.

One patient with polyostotic fibrous dysplasia had a subsequent fracture of the ipsilateral shaft femur, which was managed on expectant lines. The proximal femur is one of the common locations for benign lesions in children and adolescents, and these include varied diagnoses like a simple bone cyst, aneurysmal bone cyst, fibrous dysplasia, non-ossifying fibroma, and eosinophilic granuloma. The symptomatic ones usually present with pain and limp. The lesions mandate surgical management when they are large enough (involvement of >2/3rd cortex), affecting the bony stability [5]. This study aimed to present the treatment results of lytic lesions of the proximal femur through an anterolateral approach using curettage and non-vascularized fibular grafting in children. Recent data continue to emphasize the clinical burden of these lesions in the pediatric population. In a 2024 retrospective series of 41 children with benign proximal femoral cysts, pathological fractures were reported in 32% of cases and recurrence after curettage and filling in 17.1% at a mean follow-up of 26.5 months[9]. Likewise, the 2023 multicentre European Paediatric Orthopaedic Society (EPOS) study on primary aneurysmal bone cysts of the proximal femur in children and adolescents documented failure rates of 36-43% for both open and percutaneous interventions and underscored the elevated risk of pathological fractures in this region [10]. These findings highlight the therapeutic challenges that persist despite advances in technique and support the need for approaches that allow stable fixation and biological healing, such as fibular grafting through the anterolateral route. The anatomical location of these lesions usually extends from the femoral neck, sometimes close to the proximal femoral growth plate, to the sub trochanteric region. Plain radiographs and advanced imaging (CT and MRI) help precisely map the extent and sometimes nature of the lesion. Surgical management includes a reliable diagnosis, curettage, filling of the defect and prevention/ stabilization of a pathological fracture. Creating a cortical window is required for an effective curettage of the lesion. The use of high-speed burr in cases of aggressive lesions like aneurysmal bone cysts to extend the surgical margins beyond the reactive margin is considered essential to prevent recurrence [11]. The various biomaterials for such a reconstruction include autografts, allografts and synthetic bone substitutes. Among autografts, fibula is a choice among many surgeons due to its structural properties and minimal donor site morbidity [12]. It carries with it the additional advantage of osteoinductive properties [13]. The structural properties prevent a pathological fracture and the collapse of the femoral head [14]. In contrast, allografts carry a high risk of infection, fracture, nonunion and delayed union [15]. Also, due to their processing, the allografts have reduced osteogenic and osteoconductive properties and the risk of disease transmission [16]. Although synthetic bone substitutes, with their osteoconductive properties, are not at risk of disease transmission, and their quantity may not be limited, their use is limited by their insufficient structural strength and the lack of osteoinductive properties [17,18]. In this regard, using fibula as a reconstructive material is apt. Its advantage lies in its being expandable with a straightforward surgical approach [13,14]. Unlike bone cement, it is a biological material incorporating a natural osseous structure. The mechanical support provided by the cortical bone of the fibula is an added advantage [19]. Literature also suggests that periosteum preserving fibula grafting leads to regeneration of neo-fibula as early as six months in 66% of cases. In the remaining, the non-continuous regeneration had no clinical implication [20]. The anterolateral approach was used in this study to reach the proximal femur and create a window for curettage, which comes with its advantages. The anterolateral approach to the hip is one of the most typical approaches used for the hip, and it provides good and wide visibility into the lesion while performing curettage [7]. The planes in this approach are inter-muscular and do not involve detachment of any muscles from their origin or insertion, as is needed in the lateral approach. This leads to faster healing in the post-operative period. With the preservation of the femoral lateral cortex in the anterolateral approach, the familiar implants commonly used in the proximal femur, like a cannulated cancellous screw or a dynamic hip screw with a plate, can be used, if needed. This study included 14 males with a mean age of 7.1 years. An aneurysmal cyst was the most common lesion noted. It is imperative to rule out a prior pathological fracture as well, and enough care should be taken to prevent an intra-operative fracture. A pathological fracture has the potential to alter the course of the treatment as well as the disease. The results of our study demonstrated that the average radiological healing time post-grafting for these lesions was approximately 4 months. Satisfactory hip scores were demonstrated at follow-up, indicating a pain-free, weight-bearing and ambulatory child. Also, regular and close follow-ups should be obtained to detect implant loosening early. This is one of the first studies to demonstrate the results of benign lytic lesions of the proximal femur in children using an anterolateral approach to the lesion site and fibular autograft. A single surgeon performed all the surgeries. Its retrospective nature and sample size limits the results of this study. The heterogeneity of the various diagnoses could also have limited the results.

## Conclusion:

The anterolateral approach to the hip, combined with non-vascularized fibular grafting, emerged as a viable option for managing lesions of the proximal femur in children. The technique offers diagnosis and healing within the same surgical intervention. The approach permits the use of lateral cortex-based implants if the need arises. The use of the grafts hastened healing and final follow-up revealed good clinical function.

## Advancement to knowledge:

Demonstrate a reproducible anterolateral approach with fibular autografting that preserves the lateral cortex and allows implant use in pediatric proximal femoral lesions. Provide evidence of reliable early healing (∼4 months) with good functional outcomes, addressing the high fracture and recurrence risk reported in recent literature. Highlighted a cost-effective, biologically sound technique applicable in resource-limited settings while maintaining structural stability and clinical recovery.

## Statement and declarations:

## Funding:

The authors declare that no funds, grants, or other support were received during the preparation of this manuscript.

## Competing interests:

The authors have no relevant financial or non-financial interests to disclose.

## Ethics approval:

This study was performed in line with the principles of the Declaration of Helsinki. Ethical approval was obtained for the study from the IEC of the site of study (E.1/IEC/CNBC/04/02/2019/6869).

## Consent to participate:

Written informed consent was obtained from the parents.

## Consent to publish:

Written informed consent was obtained from the parents.

## Author contribution:

All authors contributed to the study conception and design. Material preparation, data collection and analysis were performed by Dr Aditya, Dr Anil, Dr Lokesh and Dr Yogesh. The first draft of the manuscript was written by Dr Sitanshu and Dr Varun and all authors commented on previous versions of the manuscript. All authors read and approved the final manuscript.

## Availability of data and materials:

NA

## Code availability:

NA

## Preprint:

No PREPRINT has been submitted.

## Funding:

None received

## Figures and Tables

**Table 1:** Patient details included in the study (n=14

**S.No.**	**Age(In Years)**	**Gender**	**Diagnosis**	**Implant Used**	**Timing Of Radiological Union (Months)**	**Final Follow Up Period (Years)**	**Harris hip score at final follow up**	**Additional Procedures**	**Complications**
1.	8	M	Aneurysmal bone cyst	Nil	3	4	84	Nil	None
2.	4.7	M	Non-ossifying fibroma	Nil	2	2	72	Nil	None
3.	9.6	M	Eosinophillic granuloma with aneurysmal bone cyst	Cannulated cancellous screws	3	6	88	Implant Removal	Loosening of screw
4.	5.7	M	Aneurysmal bone cyst	Rush rods	4	2.5	78	Nil	Presented with pathological fracture
5.	5.1	M	Fibrous dysplasia	Rush rods and cannulated cancellous screws	6	3	82	Implant Removal	None
6.	7.5	M	Non-ossifying fibroma	Nil	4	6	78	Revision Surgery	None
7.	8.1	M	Fibrous dysplasia	Nil	6	2.5	78	Nil	None
8.	12.5	M	Aneurysmal bone cyst	Dynamic hip screw (subtrochanteric fracture)	8	6	74	Nil	None
9.	7.1	M	Aneurysmal bone cyst	Nil	3	2	92	Nil	None
10.	5.9	M	Tubercular osteomyelitis	Nil	4	2	70	Nil	None
11.	6.4	M	Polyostotic fibrous dysplasia with fracture neck of femur	Cannulated Cancellous Screws	3	2	68	Implant Removal	Ipsilateral Fracture Shaft Femur
12.	3.1	M	Aneurysmal bone cyst	Nil	4	3	78	Nil	None
13.	11.7	M	Aneurysmal bone cyst	Nil	3	2	80	Implant Removal	None
14.	5.1	M	Aneurysmal bone cyst	Nil	3	2	82	Nil	None

## References

[1] Zekry KM (2018). J Orthop Surg Res..

[2] Zhao Z (2025). Front Oncol..

[3] https://www.ncbi.nlm.nih.gov/books/NBK532982/.

[4] Bloem JL (2012). Journal of Radiology..

[5] Kundu ZS (2013). Arch Orthop Trauma Surg..

[6] Mittal R, Banerjee S. (2012). J Clin Orthop Trauma..

[7] Schneidmueller D (2021). Oper Orthop Traumatol..

[8] Agarwal A (2023). J Pediatr Orthop B..

[9] Li T (2024). Front Pediatr..

[10] vangeloven TPG (2023). Journal of Pediatric Orthopaedics..

[11] Dormans JP (2004). Clin Orthop Relat Res..

[12] Arai K (2002). Plastic and Reconstructive Surgery..

[13] Korompilias AV (2011). Microsurgery..

[14] Kainth GSJ (2011). Orthop Surg (Hong Kong)..

[15] Ferrari S (2005). JCO..

[16] Wisanuyotin T (2022). Sci Rep..

[17] Sanmartin De (2018). J Int Med Res..

[18] Gorski S.M (2017). Biomaterials..

[19] Gorski SM (2022). Anticancer Research..

[20] Agarwal A, Kumar A (2016). International Orthopaedics (SICOT)..

